# Chronic Active Epstein–Barr Virus Infection: The Elucidation of the Pathophysiology and the Development of Therapeutic Methods

**DOI:** 10.3390/microorganisms9010180

**Published:** 2021-01-15

**Authors:** Ayako Arai

**Affiliations:** 1 Division of Hematology and Oncology, Department of Internal Medicine, St. Marianna University School of Medicine, 2-16-1 Sugao, Miyamae-ku, Kawasaki, Kanagawa 216-8511, Japan; ara.hema@marianna-u.ac.jp; Tel.: +81-44-977-8111; Fax: +81-44-977-8361; 2 Department of Hematological Therapeutics, Graduate School of Medical and Dental Sciences, Tokyo Medical and Dental University (TMDU), Bunkyo-ku, Tokyo 113-8510, Japan

**Keywords:** systemic chronic active Epstein–Barr virus infection, cutaneous chronic active Epstein–Barr virus infection, EBV-positive T-cell lymphoma of childhood, WHO classification

## Abstract

Chronic active Epstein–Barr virus infection (CAEBV) is a disease where Epstein–Barr virus (EBV)-infected T- or NK-cells are activated and proliferate clonally. The symptoms of this dual-faced disease include systemic inflammation and multiple organ failures caused by the invasion of infected cells: inflammation and neoplasm. At present, the only effective treatment strategy to eradicate EBV-infected cells is allogeneic stem cell transplantation. Lately, the investigation into the disease’s pathogenic mechanism and pathophysiology has been advancing. In this review, I will evaluate the new definition in the 2017 WHO classification, present the advancements in the study of CAEBV, and unfold the future direction.

## 1. Introduction

It is known that Epstein–Barr virus (EBV) causes B-cell lymphomas such as Burkitt lymphoma and diffuse large B-cell lymphoma, but their genome is also known to be positive in certain T- or NK-cell lymphoid neoplasms such as extranodal NK/T-cell lymphoma (ENKL), aggressive NK-cell leukemia (ANKL), and chronic active Epstein–Barr virus infection (CAEBV). As stated in the WHO 2017 classification, “The diagnosis of extranodal NK/T-cell lymphoma should be considered with skepticism if EBV is negative” [1], and tumor cells are EBV-positive in most cases of ENKL. In the case of ANKL, 85 to 100% of tumor cells are EBV-positive [2]. Because the confirmation of EBV-infected T- or NK-cells is one of the diagnostic criteria of CAEBV [3], neoplastic cells are EBV-positive in all CAEBV cases. 

CAEBV has been reported as a rare disease mainly in Japan and other East Asian countries. Since the WHO classification of tumors of hematopoietic and lymphoid tissues revised in 2017 (WHO 2017) defined CAEBV as a T- or NK-cell neoplasm, attention and reports on the disease have gradually increased worldwide [3]. Based on the diagnostic criteria coinciding with that of the WHO 2017 classification, the author and colleague researchers conducted a nationwide fact-finding survey in Japan to investigate the clinical features and treatment strategies of CAEBV. The results of this survey were reported and published in 2020 [4]. The aims of this review are to summarize the results of the survey and to raise issues on the WHO 2017 classification, citing from the survey report.

## 2. What Is CAEBV?

CAEBV is an intractable and progressive disease, the symptoms of which include persistent or recurrent inflammation, harboring EBV-infected clonally proliferating T- or NK-cells. At first, the disease was considered sustained infectious mononucleosis (IM), and it was named “chronic active Epstein–Barr virus infection” [5]. However, in 1988, Jones et al. reported cases of EBV-infected T-cells that were clonally proliferating [6]. After this, reports indicating CAEBV as a neoplastic disorder followed, mainly from Japan [7,8]. Moreover, it was discovered that two diseases of unique cutaneous symptoms, hydroa vacciniforme-like lymphoproliferative disorder (HV-LPD) and severe mosquito bite allergy (sMBA), show similar progress as CAEBV. They manifest EBV infection of T- or NK-cells with their clonal proliferation and develop lymphoma during the course. In 2012, Kimura et al. proposed to unify the classification of CAEBV, HV-LPD, and sMBA as EBV-positive T- or NK-lymphoproliferative diseases (EBV-T/NK-LPDs) [9]. Considering these evolvements, EBV-T/NK-LPDs were explicitly described as EBV-positive T- or NK-cell lymphoma in the WHO 2017 classification [3].

## 3. The Definition and the Diagnostic Criteria of CAEBV in the WHO 2017 Classification

Although it has been named chronic active EBV “infection,” the condition is actually a neoplasm. In the WHO 2017 classification, eight diseases are described as EBV-T/NK-proliferations, and the diseases are accompanied by EBV-infected T- or NK-cell proliferation (Table 1) [3]. Among them, four diseases, namely systemic CAEBV (sCAEBV), HV-LPD, sMBA, and systemic EBV-positive T-cell lymphoma of childhood, are grouped as EBV-T/NK-LPDs, according to the proposal by Kimura et al. [9]. In the explanation of EBV-T/NK-LPDs, sCAEBV is defined as a disease of persistent inflammation with proliferating EBV-positive T- or NK-cells. The proliferation patterns of EBV-infected cells are diverse from polyclonal, oligoclonal, to monoclonal, and the degree of inflammatory symptoms also vary. The diagnosis criteria are to include all the following conditions from (1) to (4); (1) IM-like symptoms persisting for > 3 months, (2) increased EBV-DNA (>10^2.5^ copies/μg) in peripheral blood (PB), (3) histological evidence of organ disease, and (4) demonstration of EBV RNA or viral protein in affected tissues in patients without known immunodeficiency, malignancy, or autoimmune disorders. Based on our nationwide survey, the main symptoms and signs of sCAEBV in diagnosis were: fever (85%), hepatosplenomegaly (70%), lymphadenopathy (53%), cardiac dysfunction (9%), aneurysm (9%), gastrointestinal symptoms (8%), neurological symptoms (8%), vasculitis (7%), uveitis (4%), and HLH (26%). The main laboratory findings were neutropenia (16%), anemia (14%), thrombocytopenia (30%), high ALT (31%), and high sIL-2R (27%) [4]. Because sCAEBV shows various clinical features, patients may first visit any department in hospitals. Every clinician should have sufficient knowledge of CAEBV to be able to doubt the disease, regardless of one’s specialty field. 

There are two disease types of cutaneous CAEBV: HV-LPD and sMBA. HV-LPD is a disease mostly of children, mainly associated with vesicles that develop in sun-exposed skin areas, leaving scars after healing [10]. The disorder varies its form from a kind that remains local to the skin to a kind that progresses to fever, lymphadenopathy, or hepatosplenomegaly. sMBA is a disorder associated with mosquito bites followed by fever accompanied by redness, vesicles, ulcers, and necrosis, leaving scars in the areas of bites [11,12]. The WHO 2017 classification specifies that HV-LPD and sMBA are the conditions of which the lesions are limited to the skin [3]. Although the WHO 2017 classification mentions the proliferation of only EBV-infected NK-cells in the lesioned area of sMBA, there are also cases of infected T-cells [4,9]. 

Systemic EBV-positive T-cell lymphoma of childhood is defined as “a life-threatening illness of children and young adults, characterized by a clonal proliferation of EBV-infected T-cells with an activated cytotoxic phenotype.” According to the WHO 2017 classification, there are cases of pathogenesis in the primary infection of EBV or cases of CAEBV progression [3]. Many cases accompany hemophagocytic lymphohistiocytosis (HLH), and the clinicopathological features of some cases overlap with those of ANKL. The tissue invasion of small lymphocytes without atypia is its pathological feature. There are also cases of pleomorphic cells, large-sized cells, karyokinesis, and phagocytosis in the liver and the spleen.

## 4. The Relationship between ENKL and CAEBV

ENKL is a neoplasm of NK- or T-cells, and the EBV genome is positive in neoplastic cells [1,13]. The regions of ENKL usually exist in the extranodal area, and the most infected site is in the upper aerodigestive tract, including the nasal cavity, nasopharynx, paranasal sinuses, and palate. The disease is often accompanied by inflammatory symptoms, and it is sometimes difficult to distinguish ENKL from CAEBV. Furthermore, ENKL can develop from CAEBV during the course [9]. For a differential diagnosis of the two diseases, I recommend checking the following three points. The first point is where the localization of EBV-DNA in PB occurs. In ENKL, EBV-DNA is mainly detected in plasma [14]. On the other hand, it is detected dominantly in mononuclear cells in PB in the case of CAEBV [15]. Second, verify if the images of FDG-avid in 2-deoxy-2-[18F]fluoro-D-glucose (FDG-PET/CT) are different. The lesions of ENKL are highly FDG-avid, whereas those of CAEBV do not show an elevated uptake of FDG [16]. Thirdly, it is important to examine the clinical course. In a patient with sustained inflammatory symptoms or characteristic skin symptoms such as HMB or HV for more than 3 months, one should suspect that CAEBV may exist in the background. It is indispensable to distinguish these two diseases because their treatment strategies are totally different. The outcome of ENKL treated with chemotherapies, with or without irradiation, has recently been dramatically improved [17]. However, CAEBV has been chemotherapy resistant, and allogeneic hematopoietic transplantation is essential to cure [18].

## 5. The Differences between the Diagnostic Criteria of Japan and the WHO 2017 Classification

In 2015, a research group of Measures Against Intractable Diseases of the Ministry of Health, Labour and Welfare of Japan (MHLW research group), created and published the diagnostic criteria of CAEBV (Table 2) before the WHO 2017 classification was published [19]. MHLW research group defines CAEBV as a disease characterized by IM-like symptoms and systemic inflammation such as fever, lymphadenopathy, hepatosplenomegaly, an increased amount of EBV genome in PB or in lesion tissue, and EBV infection of T- or NK-cells. According to MHLW research group, HV-LPD and sMBA are not to be diagnosed as CAEBV. sCAEBV defined in the WHO 2017 classification falls under this category.

In this review, I will compare the Japanese diagnostic criteria against that of the WHO 2017 classification. The definition of CAEBV, which reads as “an EBV-infected T- or NK-cell neoplasm associated with chronic inflammation,” is the same in both guidelines. However, their diagnostic criteria differ slightly. The major difference is the methods to identify infected cells. Both require the identification of EBV-infected T- or NK-cells. Whereas the WHO 2017 classification demands the identification of EBV-encoded small RNA by in situ hybridization (EBER-ISH) or EBV protein such as LMP1 and EBNA1 using pathological specimen, the Japanese criteria do not limit the methods to EBER-ISH or viral protein detection. As mentioned in the footnote of Table 2, the Japanese guideline also allows the detection of EBV infection on separated T- or NK-cells from PB by flow cytometry or antibody-conjugated magnetic beads sorting. The reason for allowing these methods is due to the difficulty of biopsy in the cases of sCAEBV. sCAEBV does not manifest solid tumors. Additionally, many patients indicate thrombocytopenia and vascular lesions. Therefore, biopsy is usually challenging. According to the Japanese nationwide survey, only 15% of all the cases succeeded in diagnosing CAEBV by histology [4]. All other cases diagnosed CAEBV by detecting EBV-DNA or EBNA in separated T- or NK-cells from PB. In CAEBV, a large number of EBV-infected cells are detectable in PB. The advantages of analysis using PB are that it involves minimal invasion and reveals the infection in multiple phenotypes of lymphocytes through more detailed examination. The identification of EBV-infected cells using PB contributes to a speedy and accurate diagnosis of the disease. We hope the method will be more publicized and applied worldwide.

## 6. The Nationwide Investigation of Clinical Treatment of sCAEBV in Japan Based on the WHO 2017 Definition

**Figure 1:**
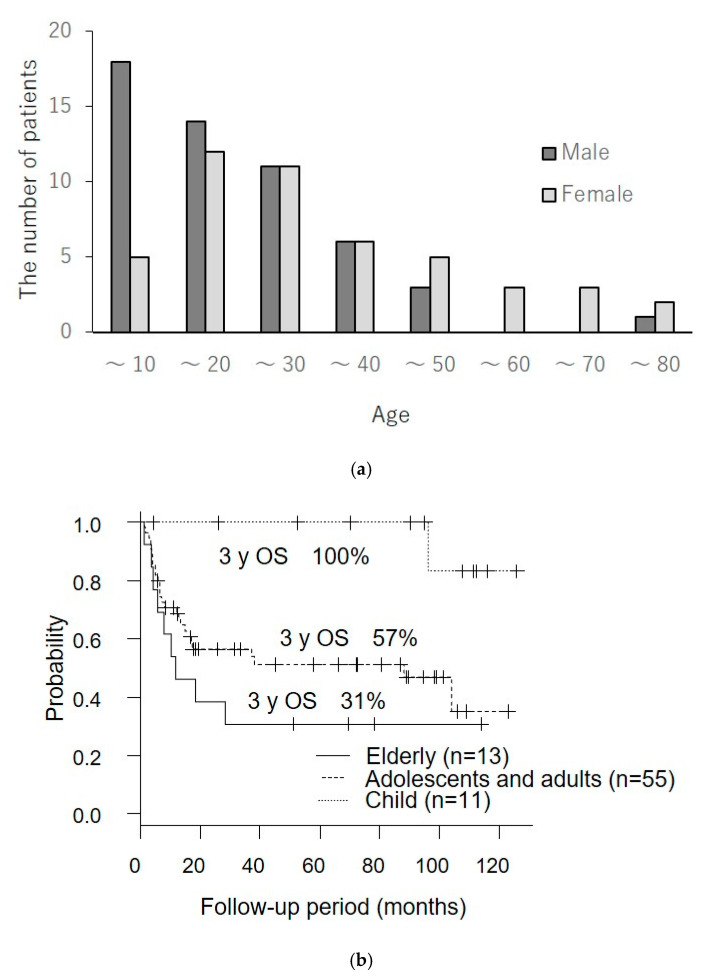

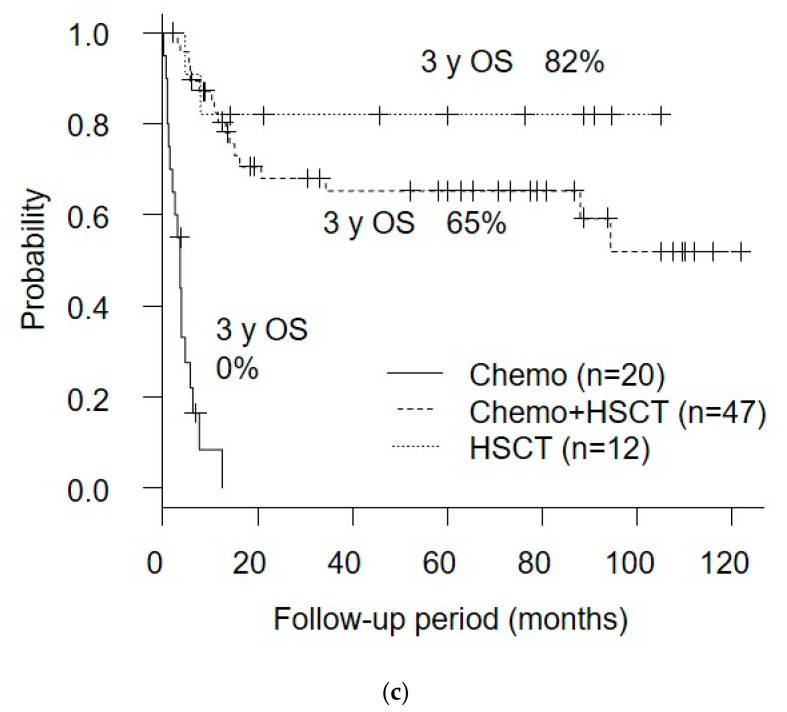
The results of the nationwide fact-finding research of chronic active Epstein–Barr virus infection (CAEBV) treatment in Japan [4]. (**a**) Sexual specificity by age; (**b**) survival rate by age; (**c**) survival rate by treatment method.

The nationwide survey of sCAEBV in Japan by the author and colleagues is the first large-scale study of sCAEBV conducted after the publication of the WHO 2017 classification [4]. The summary of the survey is as follows. Out of 100 cases analyzed, 53 were of male patients and 47 were of female patients. The age of patients ranged from 1 to 78 years, and the median was 21. Interestingly, the majority of the childhood age group below 10 were male, and the elderly age group above 45 were female (Figure 1a). The survival probability rate of the elderly age group showed a poorer prognosis compared to children (Figure 1b). The survival probability rate by the treatment method is shown in Figure 1c. The three-year survival rate in the cases treated by hematopoietic stem cell transplant (HSCT) only was 82%, that of chemotherapy followed by HSCT was 65%, and that of chemotherapy only was a tough result of 0%. There was no case that successfully eliminated EBV-infected T- or NK-cells by drug therapy using steroids, immunosuppressive agents, and other chemotherapies.

The results of the survey suggest a possibility of CAEBV in childhood and in the elderly are different disorders, although their clinical features are common. I expect these findings will contribute to elucidating the disease pathophysiology. Furthermore, the investigation affirmed once again that there is no effective drug, and HSCT is the only treatment to attain a complete cure. Developing a drug remains to be an urgent issue.

## 7. The Issues on the WHO 2017 Classification, Which Surfaced after the Nationwide Investigation

Here are my personal opinions on the WHO 2017 classification. As a clinician heavily involved in CAEBV, I highly value the fact that the disease is now clearly identified as a kind of lymphoid neoplasm. On the other hand, I think there are several problems to be solved. The first is the positioning of histology in diagnosis. A detailed analysis of lesion tissue is essential in elucidating the pathophysiology of CAEBV. However, the severe conditions of patients make biopsy difficult, especially when demonstrating abnormal blood coagulation. Therefore, the analysis of infected cells in PB should be reviewed and verified as another option in the diagnosis criteria. New methods are becoming available to make analysis easier. The detection of EBV-DNA in each lymphocyte fraction isolated by antibody-conjugated magnetic beads is effective to determine the phenotypes of EBV-infected cells. Recently, a new flow cytometry technique combining surface marker staining with in situ hybridization for EBER RNAs has been developed [20,21]. Second, I want to argue the disease definition of systemic EBV-positive T-cell lymphoma of childhood. According to the WHO 2017 classification, one can interpret it as a disease associated with a rapid progression of systemic inflammation or HLH, accompanying EBV infection of T-cells and its monoclonal proliferation. The WHO 2017 classification further describes that systemic EBV-positive T-cell lymphoma of childhood includes both severe state of IM and rapidly progressive CAEBV. However, is it appropriate to group these two conditions together as the same disease? Another problem is that the difference between systemic EBV-positive T-cell lymphoma of childhood and sCAEBV is not clear. To answer these questions, we must accumulate numbers of cases and further examine the details. Lastly is the disease definition of EBV-HLH. The WHO 2017 classification does not mention its definition, but EBV-HLH is generally described as a HLH positive for EBV-DNA in PB. The disease defined in the WHO 2017 classification likely includes the cases of HLH developing from both primary infection of EBV and EBV-positive neoplasms: ENKL, ANKL, and CAEBV. The former cases are severe IM and may have a background of immunodeficiency. I believe that EBV-HLH associated with primary infection is to be specified separately from HLH which develops from EBV-positive lymphoma. 

## 8. The Treatment of CAEBV

Although most CAEBV patients show a chronic course of progress by months or by years, the prognosis is poor if not treated properly. According to the study by Kimura et al. of 108 cases, of which the age of patients ranges from 1 to 50 years old with the median observation period of 46 months, 44% of the cases resulted in death due to severe organ dysfunction [9]. Whereas the 15-year-overall survival in the group of onsets under the age of 8 years was 59.7%, the rate was significantly lower at 27% in the group of onsets above 8 years of age. There are other similar reports including ours, and we can assume that the higher age of onset is one of the factors of poor prognosis [4,22].

Because CAEBV has two characteristics of inflammation and tumor, the treatment aims to control both. Once lymphoma or HLH develops, a patient follows a fatal course of progress. Treatments should start before the development of such disorders. The combination use of steroids, etoposide, and cyclosporine or cytotoxic chemotherapies, in accordance with the treatment of lymphoma, has been chosen [23], but there is no effective chemotherapy to dissipate EBV-DNA load in PB, which means to eradicate EBV-infected neoplastic cells [4]. 

There are reports on reduced-intensity conditioning HSCT that achieved the elimination of EBV-infected neoplastic cells [24,25]. Three reports revealed poor prognoses for the cases showing disease activities before starting conditioning treatment [4,9,25]. The disease activities are characterized by persistent inflammatory symptoms: fever, increase in ALT to above double the upper limit value of each institution’s standard twice consecutively, progressive cutaneous lesion, vasculitis, and uveitis. Therefore, today’s role of chemotherapy is to improve the outcome of HSCT by controlling disease activities at the time of HSCT.

## 9. The Elucidation of Pathogenesis Mechanisms and the Development of a Treatment Method

EBV is ubiquitous, and the majority of human beings are infected before reaching adulthood. Why do only some human beings develop CAEBV? The mechanism of CAEBV development is gradually being unraveled.

How does EBV infect T- or NK-cells? It is known that CD21 function as infectious receptors when EBV infects B cells [26], but CD21 is also slightly expressed in T-cells [27]. There is also a report of CD21 expression in NK-cells caused by trogocytosis, an immunological synapse that occurs when EBV contacts B cells, resulting in possible EBV infection [28]. A similar mechanism also exists in T-cells [29]. In addition, some reports point out that the infection of EBV in T- and NK-cells occurs during the acute phase of IM, and one can assume that infection is possible [30,31]. There may be an immunological disturbance in CAEBV patients that allows persistent infection.

It was reported that cytotoxic T-cells (CTL) decreased in number or showed dysfunction in CAEBV [32,33]. Additionally, some congenital immunosuppressive disorders, such as the case of autoimmune lymphoproliferative disorder (ALPS) with *FAS* gene mutation [34], *IL2RG* gene mutation [35], or the case of perforin mutation [36], can be complicated by CAEBV-like conditions. Undetermined immunosuppressive disorders may exist in the background. 

Tumorigenicity of EBV is also drawing attention. In vitro EBV infection in T- or NK-cell lines suppresses serum-depletion- or anti-cancer-reagent-induced apoptosis of infected cells. It was reported that the constitutive activation of NF-kB was induced by in vitro EBV infection [37,38]. The inhibitors of NF-kB induce apoptosis of EBV-positive T- or NK-cells from CAEBV. These results indicate that EBV infection itself renders T- or NK-cells immortal. Our group previously reported that in vitro EBV infection also induces P-glycoprotein in T- or NK-cells and can cause chemotherapy resistance [39]. P-glycoprotein is an energy-dependent efflux pump and excretes drugs from the cytoplasm, resulting in low-intracellular drug concentrations and poor sensitivity to chemotherapy. Furthermore, a high level of activation-induced cytidine deaminase (AID) expression was reported in CAEBV [40]. AID is a protein that actively carries out gene mutation and is indispensable in somatic hypermutation and class switch recombination of immunoglobulin genes. Constitutively expressed AID acts as a genomic mutator, leading to the development of B-cell lymphoma [41]. One can assume that EBV infection induces AID expression in B cells [42]. There are reports of enhanced AID expression in chronic infectious diseases-based malignancies such as Burkitt lymphoma, which is an EBV-positive B-cell lymphoma, *Helicobacter-Pylori*-positive gastric cancer, and hepatitis-C-virus-positive hepatocellular cancer [43]. Thus, we can hypothesize that EBV induces gene mutation through AID expression in T- or NK-cells. Further study is expected to validate the hypothesis. 

Some investigators reported the use of next-generation sequencing. Okuno et al. performed whole-exome sequencing (WES) on T-, B-, and NK-cell subsets from CAEBV patients [44]. They reported that the most frequently mutated gene was *DDX3X*, an RNA helicase gene, detected in 16% (14/83). They also reported that the overall survival (OS) of the patients carrying *DDX3X* mutation at diagnosis was significantly shorter compared to the patients without the mutation. Interestingly, Jiang et al. performed WES on tumor cells from ENKL and reported that *DDX3X* frequently mutated (20%, 21/105) [45]. They determined that the mutation exhibited growth-promoting effects on NK-cells in comparison to wild-type protein. *DDX3X* mutation was also detected in Burkitt lymphoma. Many reports focus on *DDX3X* and their association with cancers. The mutation may be a common driver of EBV-positive neoplasms. However, Okuno et al. detected other various mutations aside from *DDX3X* in CAEBV: *KMT2D* (4.8%), *BCOR*/*BCORL1* (3.6%), *KDM6A* (3.6%), and *TET2* (2.4%). They reported the detection rate of at least one somatic mutation in CAEBV by WES was 52% as a whole. These findings indicate a diversity of the background in CAEBV. 

Is there a unique attribute to the viruses of CAEBV patients? Okuno et al. conducted a genome analysis of 77 CAEBV patients’ infected cells and reported that, in 27 cases (35%), they discovered deficiencies common to other EBV-positive neoplasm-infected cells [44]. Among these cases, 14 observed the deficiencies in the parts that code microRNA (miR-BARTs), which are called BamHI A rightward (BART). miR-BARTs take part in controlling the expression of viral lytic proteins, such as BZLF1, BRLF1, and LMP1. They observed the leaky expression of the proteins by the deletions of miR-BARTs. Murata et al. suggested to name the increased expression of viral lytic genes as “prelatent abortive lytic state” [46]. The significance of this state in CAEBV development needs to be elucidated. In addition, cytokines and chemokines associated with inflammation are possible targets of miR-BARTs. The upregulation of cytokine production by deletion of miR-BARTs can be a cause of persistent inflammation in CAEBV. We anticipate for an analysis of miR-BARTs how they develop CAEBV. Meanwhile, the CAEBV patients’ viruses in the rest of 50 cases (65%) did not show any distinctive genome abnormality. The result hints at the possibility of host factors involved in their development.

In 2018, the author and colleagues discovered that the transcription factor of STAT3 constitutively activates in the EBV-infected T- and NK-cells of CAEBV patients, and ruxolitinib, a JAK1/2 inhibitor, suppresses the proliferation and the inflammatory cytokine production in the cells [47]. Ruxolitinib is a worldwide approved drug for the indications of myelofibrosis and polycythemia vera. In January 2019, a group led by the author started an investigator-initiated clinical trial to verify the efficacy of ruxolitinib against CAEBV, aiming to expand the indications of this drug. We hope to improve the outcomes of HSCT, especially by its anti-inflammatory effect to control the disease activities.

## 10. Prospect

Thanks to the statement in the WHO 2017 classification, CAEBV started to attract global attention, and we expect the number of case reports to increase. At the same time, the statement generated new questions. To answer these questions, we must accumulate the number of cases and their analysis, especially of prospective studies. Due to its rarity, we cannot waste any case of CAEBV. In Japan, MHLW research group established a CAEBV registry in the National Center for Child Health and Development of Japan. It is a system to collect the records of clinical information and to enable prospective study. The national center not only manages the registry but also undertakes the analysis of infected cells in PB. The CAEBV registry started its operation only in Japan, but we need to further expand the fact-finding research of CAEBV pathophysiology and its treatment globally by collaborating with researchers outside Japan. There are a lot of expectations for the Japanese researchers who have been always at the global forefront of CAEBV investigation.

## Figures and Tables

**Table 1:** Classification of EBV-positive T-cell and NK-cell proliferations.

Diagnosis		Usual Patient Age Group(s)
EBV-positive haemophagocytic lymphohistiocytosis (benign, may be self-limited)		Paediatric, adolescent
EBV-positive - T- or NK-cell lymphoproliferative diseases		
	Systemic CAEBV	Paediatric, adolescent
	Cutaneous CAEBV, hydroa vaccinifo⸢me—likelymphop⸢oliferativediso⸢der	Paediatric, adolescent
	Cutaneous CAEBV, severe mosquito bite allergy	Paediatric, adolescent
	Systemic EBV-positive T-cell lymphoma	Paediatric, adolescent
Aggressive NK-cell leukaemia		Adult
Extranodal NK/T-cell lymphoma, nasal type		Adult
Nodal peripheral T-cell lymphoma, EBV-positive *		Adult

* Included within the category of peripheral T-cell lymphoma, NOS [3].

**Table 2:** Diagnostic criteria of Chronic active Epstein-Barr virus infection.

(1)	Sustained or recurrent infectious mononucleosis-like symptoms persist for more than 3 months
(2)	Elevated EBV genome load in the peripheral blood (PB) or the tissue lesion
(3)	EBV infection of T or NK cells in the affected tissues or the PB
(4)	Exclusion of other possible diagnoses: primary infection of EBV (infectious mononucleosis), autoimmune diseases, congenital immunodeficiencies, HIV, and other immunodeficiencies requiring immunosuppressive therapies or underlying diseases with potential immunosuppression
Patients who fulfilled criteria (1)–(4) were diagnosed with CAEBV	

Supplementary explanation: (1) Infectious mononucleosis -like symptoms generally include fever, swelling of lymph nodes, and hepatosplenomegaly; additional complications include hematological, gastroenterological, neurological, pulmonary, ocular, dermal, and/or cardiovascular disorders (including aneurysm and valvular disease), which have mostly been reported in patients with IM. EBV-HLH accompanied by primary infection of EBV and HV, whose symptoms are limited to those in the skin, should be excluded. Even if EBV-HLH or EBV-positive T- or NK-cell lymphoma/leukemia develops during the disease course, the original diagnosis of CAEBV does not change. (2) A standard for elevated EBV DNA load by quantitative PCR in the PB is more than 10^2.5^ copies/μg DNA. (3) For detection of EBV-infected cells, it is recommended to perform a combination analysis of detecting the phenotypes of the infected cells (immune fluorescent staining, immune histological staining, magnetic bead sorting) and detecting EBV (EBNA staining, EBV-encoded small RNA in situ hybridization, PCR for EBV DNA). (4)Patients who were diagnosed with congenital immune deficiencies, autoimmune diseases, collagen diseases; patients who were pathologically diagnosed with malignant lymphomas (Hodgkin lymphoma; extranodal NK/T-cell lymphoma, nasal type (ENKL); angioimmunoblastic T-cell lymphoma; peripheral T-cell lymphoma (PTCL); aggressive NK-cell leukemia (ANKL)); and patients who were diagnosed with an iatrogenic immunosuppressive condition, either concurrently or prior to CAEBV diagnosis, were also excluded from CAEBV [17].
